# Acute aerobic exercise modulates resting-state EEG microstate dynamics in individuals with internet addiction

**DOI:** 10.3389/fnins.2026.1802571

**Published:** 2026-04-13

**Authors:** Na Liu, Xinyi Ma, Mingrui Shao

**Affiliations:** 1 Department of Physical Education, Shanghai Maritime University, Shanghai, China; 2 School of Physical Education, Shanghai Normal University, Shanghai, China; 3 School of Physical Education, Shaanxi Normal University, Xi'An, China

**Keywords:** aerobic exercise, EEG, internet addiction, microstate, undergraduate student

## Abstract

Internet addiction (IA) is associated with impaired cognitive control and altered large-scale brain dynamics. Electroencephalogram (EEG) microstates provide a sensitive index of rapid neural network organization; however, whether acute aerobic exercise can modulate abnormal microstate dynamics in individuals with IA remains unclear. Forty young adults [IA: *n* = 20; healthy controls (HC): *n* = 20] completed resting-state EEG recordings before and after a single 30-min bout of moderate-intensity aerobic cycling. Microstate duration, occurrence, contribution, and transition probabilities were analyzed using mixed-design repeated-measures with time (pre/post) and Microstate (A–D) as within-subject factors and group (IA/HC) as a between-subject factor. Spearman correlations examined associations between exercise-induced microstate changes and IA severity. For microstate duration, the IA group exhibited longer duration of Microstate A than HC group at baseline (*t* = 2.47, *p* = 0.018). IA group showed reduced Microstate D occurrence at baseline compared with HC (*t* = 4.23, *p* < 0.001), followed by a significant post-exercise increase (*t* = −4.23, *p* = 0.001), eliminating group differences. Microstate contribution showed a significant Time × Group interaction [*F*_(1, 38) =_ 4.68, *p* = 0.037, η^2^ = 0.110], with Microstate D contribution increasing selectively in the IA group (*t* = −3.71, *p* = 0.001). Changes in Microstate D occurrence were negatively correlated with IA severity (ρ = −0.55, *p* = 0.012). A single session of aerobic exercise rapidly normalizes aberrant microstate dynamics in IA, particularly within Microstate D, highlighting exercise as an effective acute neuromodulatory intervention.

## 1 Introduction

The ubiquitous access to the internet has profoundly reshaped modern life; however, pathological and uncontrolled internet use, commonly referred to as internet addiction, has emerged as a global public health concern, particularly among adolescents and young adults (Ye et al., 2023; Pan et al., 2020). Internet addiction is characterized by compulsive engagement in online activities, impaired self-control, withdrawal symptoms, and persistent excessive use despite negative consequences (First et al., 2023; Young, 2007). Converging theoretical models suggest that internet addiction shares core neurobiological mechanisms with substance-related and behavioral addictions, including hypersensitivity of reward-related circuits (e.g., ventral striatum), weakened prefrontal executive control, and dysfunctional conflict monitoring involving the anterior cingulate cortex (Brand et al., 2019; Love et al., 2015; Weinstein and Lejoyeux, 2020). Importantly, this impulse-control imbalance is increasingly conceptualized not only in terms of regional dysfunction, but also as aberrant coordination and temporal instability of large-scale brain networks, emphasizing the need to investigate the dynamic organization of intrinsic brain activity.

Resting-state electroencephalogram (EEG), with its millisecond-level temporal resolution, provides a powerful non-invasive approach for characterizing functional brain traits associated with internet addiction. Previous EEG studies have reported increased frontal theta activity and reduced alpha power in individuals with internet addiction, suggesting impaired cognitive control and heightened vigilance at rest (Sun et al., 2019; Kashif et al., 2021). Beyond traditional spectral analyses, EEG microstate analysis has gained increasing attention as a method for capturing the rapid temporal dynamics of large-scale brain networks. EEG microstates represent quasi-stable global scalp potential topographies lasting tens to hundreds of milliseconds, reflecting transient activations of distributed neural systems (Khanna et al., 2015; Michel and Koenig, 2018). Canonical microstate classes (A–D) have been consistently linked to distinct functional networks, including auditory, visual, salience/attention, and frontoparietal executive control networks (Metzger et al., 2024; Tarailis et al., 2024). Unlike conventional frequency-based or connectivity measures, microstate analysis directly characterizes the moment-to-moment switching behavior of large-scale brain networks, making it particularly well suited for probing fast neural dynamics underlying attentional regulation and cognitive control.

Accumulating evidence indicates that alterations in microstate parameters—such as duration, occurrence, contribution, and transition probabilities—are associated with various psychiatric and neuropsychiatric disorders, including schizophrenia, major depression, and substance use disorders (Lin et al., 2022; Rohde et al., 2020; de Bock et al., 2020). These abnormalities are often interpreted as reflecting reduced neural flexibility or maladaptive network dominance. However, despite the conceptual relevance of microstate dynamics to impulse control and attentional regulation, whether individuals with internet addiction exhibit characteristic abnormalities in resting-state microstate organization remains largely unexplored. In particular, it is unclear whether microstates associated with salience processing and executive control show altered temporal dominance or transition patterns in this population.

From an intervention perspective, aerobic exercise has emerged as a low-cost, accessible, and effective strategy for improving mental health outcomes, including depression, anxiety, and addictive behaviors (Noetel et al., 2024; Theopilus et al., 2024; Lu et al., 2025). Neurobiological studies suggest that exercise induces widespread brain effects, including increased brain-derived neurotrophic factor, modulation of dopaminergic and glutamatergic neurotransmission, and enhanced prefrontal network efficiency (Gomes-Osman et al., 2017; Abdullah et al., 2022; Vergoossen et al., 2021). Considerably less is known about the acute effects of a single bout of exercise. Acute aerobic exercise has been demonstrated to transiently enhance executive function, mood, and resting-state EEG activity in healthy populations (Chueh et al., 2021; Wheeler et al., 2020), suggesting that exercise may act as a state-dependent neuromodulatory stimulus capable of rapidly reorganizing intrinsic brain dynamics. However, whether a single exercise session can immediately modulate aberrant brain network dynamics—particularly EEG microstate organization—in individuals with internet addiction has not been systematically investigated.

Two key gaps therefore remain in the current literature. First, the resting-state microstate dynamic characteristics of individuals with internet addiction are not yet clearly defined. Second, there is a lack of direct neurophysiological evidence regarding whether a single bout of aerobic exercise can rapidly reshape abnormal brain dynamics in this population. Accordingly, the present study aimed to (1) compare resting-state EEG microstate parameters between individuals with internet addiction and healthy controls to identify trait-like abnormalities in brain dynamics, and (2) examine the acute modulatory effects of a single bout of moderate-intensity aerobic exercise on EEG microstate features in both groups. We hypothesized that, relative to healthy controls, individuals with internet addiction would exhibit aberrant microstate dynamics, particularly involving microstates linked to salience processing and cognitive control. Furthermore, we expected that a single exercise session would selectively modulate these abnormal microstate parameters in the internet addiction group, shifting their brain dynamics toward a more adaptive pattern. By integrating EEG microstate analysis with an acute exercise intervention, this study aims to provide novel insights into the rapid neural dynamics underlying internet addiction and to offer empirical support for exercise as a fast-acting, non-pharmacological neuromodulatory strategy.

## 2 Methods

### 2.1 Participants

Participants were recruited from the university student population through campus advertisements and online platforms. Participants were screened and assigned to either the internet addiction (IA) group or the healthy control (HC) group. Inclusion criteria for the IA were: (1) a score ≥50 on the Chinese version of Young's Internet Addiction Test (IAT) and a score ≥67 on the Chen Internet Addiction Scale (CIAS); (2) meeting at least five of the nine diagnostic criteria for “Internet Gaming Disorder” as defined in the Diagnostic and Statistical Manual of Mental Disorders, Fifth Edition (DSM-5), confirmed via a semi-structured clinical interview; (3) self-reporting excessive, non-essential internet use (>4 h per day) for over 12 months, resulting in significant functional impairment. The HCG inclusion criteria were: (1) scores within the normal range on both the IAT (< 40) and CIAS (< 50), with self-reported healthy internet use patterns; (2) no history of psychiatric or neurological disorders, confirmed via a brief interview. Participants in the HCG were group-matched to the IAG on age, sex, years of education, handedness (all right-handed), body mass index (BMI), and habitual physical activity level assessed by the International Physical Activity Questionnaire–Short Form. Exclusion criteria for both groups included a current or past diagnosis of any major Axis I psychiatric disorder (e.g., major depressive disorder, anxiety disorders, ADHD), neurological disease, substance abuse, any contraindication to exercise, or long-term use of psychotropic medications. All participants provided written informed consent after receiving a detailed explanation of the study procedures. Ethical approval for this study was obtained from the institutional review board of Shanghai Normal University.

### 2.2 Acute aerobic exercise intervention

**Figure 1:**
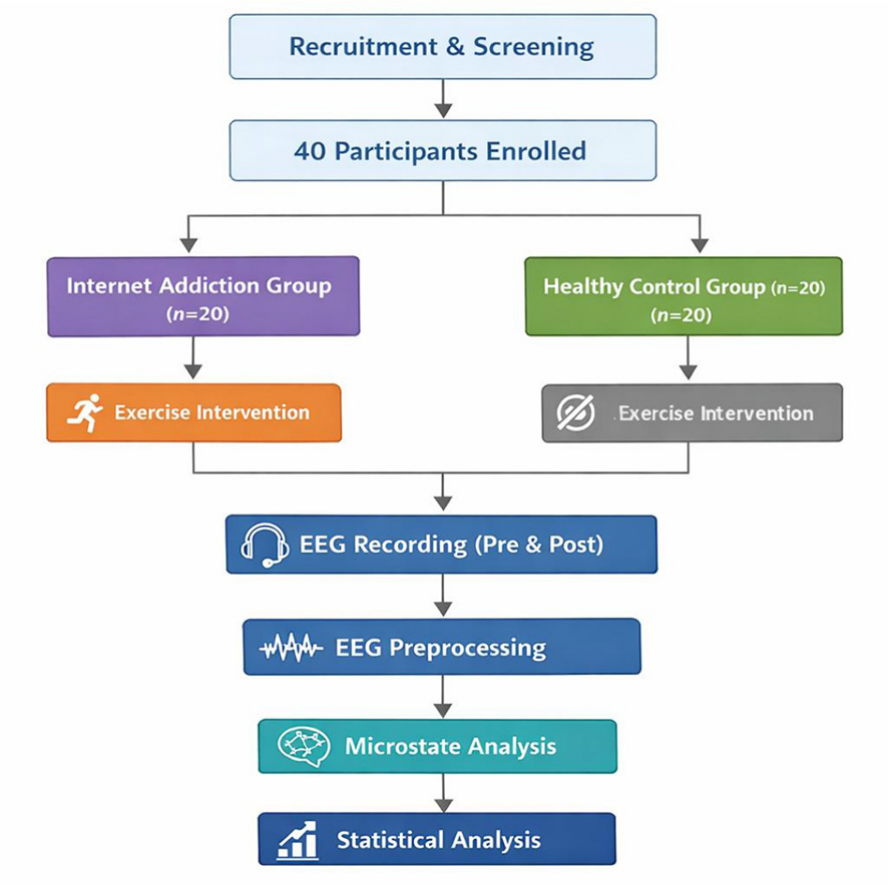
Experimental procedure of the acute aerobic exercise intervention.

All participants in the HC and IA groups completed a single bout of acute aerobic exercise under identical laboratory conditions. The overall experimental procedure is illustrated in Figure 1.

The intervention consisted of 30 min of moderate-intensity cycling performed on an electronically braked cycle ergometer. Exercise intensity was defined as 60%−70% of age-predicted maximal heart rate (HR_max = 220 – age). Heart rate was continuously monitored using a wireless chest-strap heart rate monitor (Polar H10, Polar Electro Oy, Finland), and external workload was adjusted when necessary to maintain the target intensity range. Participants were instructed to maintain a steady cycling cadence throughout the session and to refrain from any additional cognitive tasks or verbal interaction. All exercise sessions were supervised by trained research personnel, with medical staff from the university hospital present on site to monitor safety and provide immediate assistance if necessary. All participants were screened for exercise contraindications before participation.

### 2.3 EEG recording and preprocessing

EEG data were recorded using a 64-channel Brain Products EEG system (Brain Products GmbH, Germany), with electrodes positioned according to the international 10–20 system. Signals were referenced online to the linked mastoids (TP9 and TP10), and electrode impedances were maintained below 5 kΩ. EEG signals were sampled at 1,000 Hz with an online band-pass filter of 0.05–100 Hz. Offline preprocessing was conducted in MATLAB R2022a (MathWorks, Natick, MA, USA) using EEGLAB (Version R2021, San Diego, USA). Continuous EEG data were first down-sampled to 500 Hz and band-pass filtered between 1 and 40 Hz. Artifact-contaminated channels were identified and interpolated using spherical spline interpolation. Independent component analysis (ICA) was then applied to remove ocular, muscular, and cardiac artifacts, with components identified based on spatial topography, power spectral characteristics, and time-course inspection. Following artifact correction, EEG data were re-referenced to the common average and segmented into resting-state epochs. Only artifact-free epochs were retained for subsequent analyses.

### 2.4 Microstate analysis

Microstate analysis was conducted using the Microstate EEGLAB toolbox. For each participant, global field power (GFP) peaks were identified from the preprocessed resting-state EEG data, and scalp potential maps at these peaks were extracted. Group-level microstate segmentation was performed using a modified k-means clustering algorithm. Based on previous literature and cross-validation criteria, four canonical microstate classes (A–D) were selected. The resulting microstate templates were then back-fitted to individual EEG data using spatial correlation, assigning each time point to the microstate with the highest correlation. Temporal microstate parameters were computed, including mean duration, occurrence rate, contribution (percentage of total time), and transition probabilities between microstates. Self-transitions (e.g., A → A) were excluded from transition probability analyses to focus on dynamic switching behavior between distinct microstates.

### 2.5 Statistical analysis

Statistical analyses were performed using SPSS (26.0; SPSS, Inc., Chicago, IL, United States). For duration, occurrence, and contribution, mixed-design ANOVA were performed with Time (pre, post) and Microstate class (A/B/C/D) as within-subject factors and Group (HC, IA) as a between-subject factor. Four canonical microstate classes were included in these analyses. Greenhouse–Geisser corrections were applied where necessary, and effect sizes were reported as partial eta squared (η^2^_*p*_). For transition probabilities, separate mixed-design ANOVA were conducted for each originating microstate. In each analysis, transitions from one origin microstate to the remaining three microstates were treated as dependent variables, with Time (pre, post) as a within-subject factor and Group (HC, IA) as a between-subject factor. For significant main effects, *post hoc* tests were performed using the Bonferroni correction to control for type I errors. The effect size for all ANOVA analyses was quantified using partial eta squared (η), where 0.0099 indicates a small effect, 0.0588 a medium effect, and ≥0.1379 a large effect.

## 3 Results

### 3.1 Demographic characteristics of participants

The demographic characteristics of the participants are summarized in Table 1. The IA and HC groups did not differ significantly in age [*t*_(38)_ = 1.04, *p* = 0.306], sex distribution [$\chi_{\left(1\right)}^{2}$ = 1.60, *p* = 0.206], height [*t*_(38)_ = 1.45, *p* = 0.156], body weight [*t*_(38)_ = 1.39, *p* = 0.174], or body mass index [BMI; *t*_(38)_ = 1.28, *p* = 0.208], indicating good comparability between the two groups in basic demographic and physical characteristics. In contrast, a highly significant group difference was observed in IAT scores. Participants in the IA group exhibited substantially higher IAT scores compared with those in the HC group *t*_(38)_ = 17.04, *p* < 0.001, confirming the presence of moderate-to-severe internet addiction in the IA group and non-addicted internet use patterns in the HC group.

**Table 1:** Demographic characteristics of participants.

Variable	IA group (*n* = 20)	HC group (*n* = 20)	*t*-value	*p*-value
Age (years)	20.1 ± 1.5	20.4 ± 1.3	−0.68	0.502
BMI (kg/m^2^)	21.8 ± 2.3	21.5 ± 2.0	0.44	0.660
Daily non-essential Internet use (hours)	6.5 ± 1.8	2.1 ± 0.7	10.55	< 0.001
IAT score	68.3 ± 9.2	32.6 ± 5.4	15.14	< 0.001
CIAS score	75.8 ± 8.1	41.2 ± 6.3	15.33	< 0.001
PA (MET-min/week)	1,850 ± 605	1,980 ± 580	−0.71	0.484

Data are presented as mean ± standard deviation. MET-min/week was calculated based on the International Physical Activity Questionnaire–Short Form. CIAS, Chen Internet Addiction Scale; IAT, Young's Internet Addiction Test; PA, physical activity.

### 3.2 Microstate temporal parameters results

**Figure 2:**
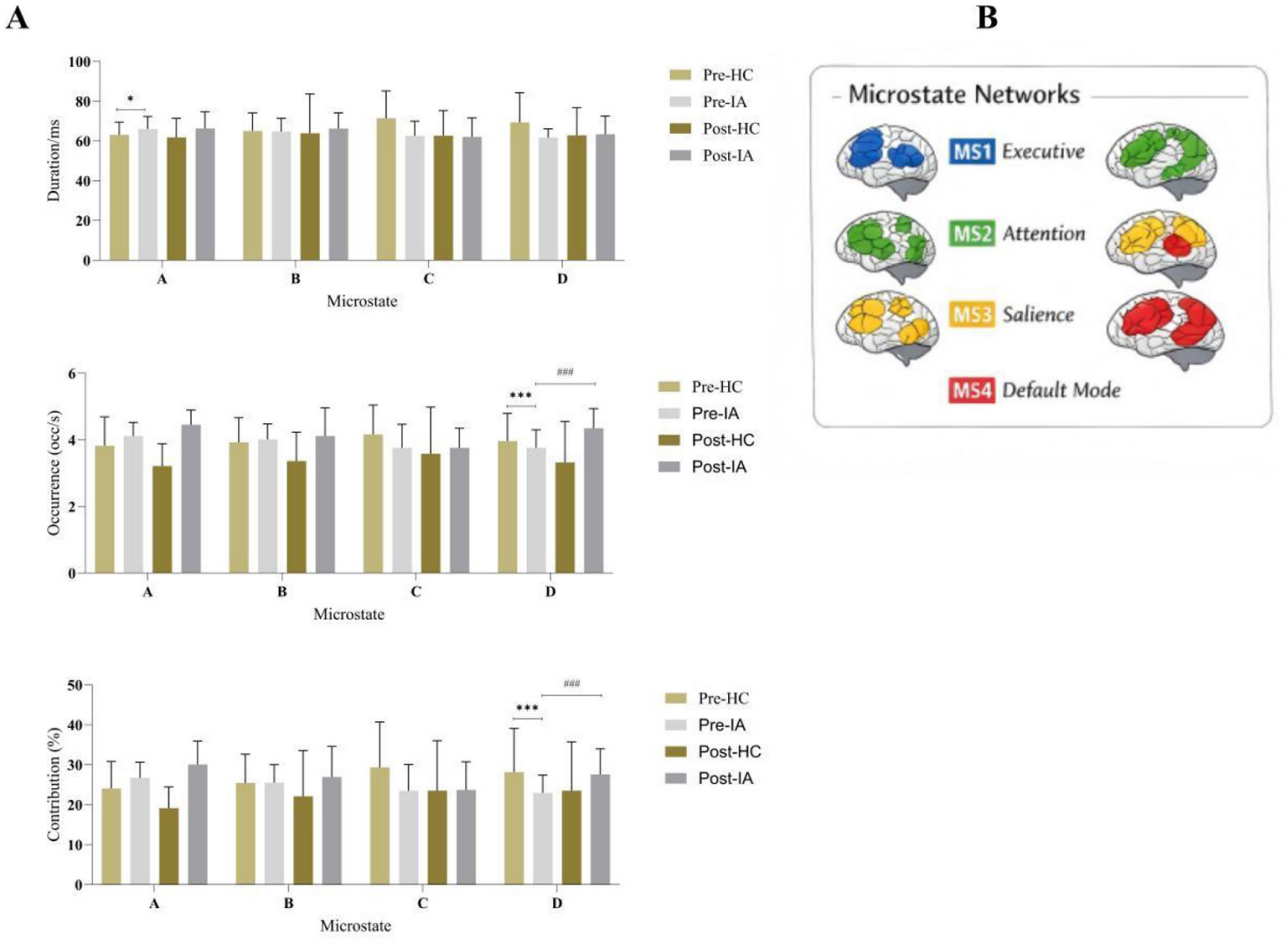
Aerobic exercise–induced modulation of EEG microstate dynamics in internet addiction. Note: Before the intervention, HC vs. IA *p < 0.05 and ***p < 0.01; After the intervention, HC vs. IA ^*###*^*p* < 0.01.

A mixed-design repeated-measures analysis of variance was conducted on microstate duration, occurrence, and contribution, with Time (pre-intervention vs. post-intervention) and Microstate type (A, B, C, D) as within-subject factors and Group (HC vs. IA) as a between-subject factor. Descriptive statistics of EEG microstate parameters for both groups before and after the aerobic exercise intervention are summarized in Table 2, and the corresponding topographic maps are shown in Figure 2B.

**Table 2:** EEG microstate parameters before and after the aerobic exercise intervention (mean ± SD).

Microstate characteristics	Microstate	Pre-intervention		Post-intervention	
		HC	IA	HC	IA
Duration (ms)	A	63.08 ± 6.41	65.00 ± 9.05	71.44 ± 13.70	69.34 ± 14.99
	B	66.01 ± 6.31	64.68 ± 6.69	62.51 ± 7.43	61.69 ± 4.43
	C	61.79 ± 9.56	63.91 ± 19.69	62.64 ± 12.63	62.75 ± 13.92
	D	66.29 ± 8.43	66.23 ± 7.90	62.03 ± 9.58	63.40 ± 9.11
Occurrence (occ/s)	A	3.82 ± 0.87	3.92 ± 0.74	4.16 ± 0.88	3.96 ± 0.83
	B	4.11 ± 0.41	4.01 ± 0.47	3.76 ± 0.71	3.76 ± 0.54
	C	3.21 ± 0.67	3.36 ± 0.87	3.58 ± 1.40	3.32 ± 1.23
	D	4.45 ± 0.44	4.11 ± 0.85	3.76 ± 0.59	4.35 ± 0.59
Contribution (%)	A	24.06 ± 6.74	25.42 ± 7.21	29.35 ± 11.34	28.13 ± 10.93
	B	26.72 ± 3.91	25.54 ± 4.49	23.48 ± 6.58	22.92 ± 4.47
	C	19.14 ± 5.29	22.11 ± 11.42	23.50 ± 12.50	23.52 ± 12.19
	D	30.08 ± 5.80	26.93 ± 7.66	23.67 ± 7.05	27.58 ± 6.38

HC, healthy control group; IA, internet addiction group. Sample size: n = 20 per group at each time point.

For microstate duration, the analysis revealed no significant main effect of time, *F*_(1, 38)_ = 0.08, *p* = 0.780, η^2^_*p*_ = 0.002. The main effect of microstate type was also not significant, *F*_(3, 114)_ = 1.82, *p* = 0.147, η^2^_*p*_ = 0.046. In addition, no significant main effect of group was observed, *F*_(1, 38)_ = 0.02, *p* = 0.892, η^2^_*p*_ < 0.001. None of the interaction effects reached statistical significance, including time × group, *F*_(1, 38) =_ 0.91, *p* = 0.347, η^2^_*p*_ = 0.023, microstate × time, *F*_(3, 114)_ = 0.17, *p* = 0.916, η^2^_*p*_ = 0.004, or the three-way interaction among time, microstate, and group, *F*_(3, 114)_ = 0.29, *p* = 0.835, η^2^_*p*_ = 0.007. The interaction between Microstate type and Group approached but did not reach statistical significance, *F*_(3, 114)_ = 2.64, *p* = 0.053, η^2^_*p*_ = 0.065. However, exploratory baseline comparisons suggested that the IA group showed a longer duration of Microstate A than the HC group, *t*_(38)_ = 2.47, *p* = 0.018, although this finding should be interpreted cautiously.

For microstate occurrence, no significant main effect of time was observed, *F*_(1, 38)_ = 0.02, *p* = 0.880, η^2^_*p*_ = 0.001. In contrast, a significant main effect of Microstate type emerged, *F*_(3, 114)_ = 10.04, *p* < 0.001, η^2^_*p*_ = 0.209, indicating robust differences in occurrence frequency across microstates. The main effect of group was not significant, *F*_(1, 38)_ = 0.30, *p* = 0.585, η^2^_*p*_ = 0.008, and none of the two-way interactions reached significance. However, a significant three-way interaction among time, microstate type, and group was detected, *F*_(3, 114)_ = 3.17, *p* = 0.027, η^2^_*p*_ = 0.077, indicating that changes in microstate occurrence across time differed between groups depending on microstate class. Follow-up analyses revealed that at baseline, the IA group showed significantly lower occurrence of microstate D compared with the HC group (*t* = 4.23, *p* < 0.001). After the intervention, paired-sample *t*-tests demonstrated a significant increase in microstate D occurrence in the IA group (*t* = −4.23, *p* = 0.001), such that post-intervention values no longer differed significantly from those of the HC group (Figure 2A). No significant pre–post changes were observed in the HC group. These follow-up findings are interpreted as microstate-specific patterns within the context of the significant three-way interaction.

Analysis of microstate contribution revealed a significant main effect of time, *F*_(1, 38)_ = 4.68, *p* = 0.037, η^2^_*p*_ = 0.110, as well as a significant time × group interaction, *F*_(1, 38)_ = 4.68, *p* = 0.037, η^2^_*p*_ = 0.110, suggesting differential temporal changes between groups. A significant main effect of microstate type was also observed, *F*_(3, 114)_ = 3.80, *p* = 0.012, η^2^_*p*_ = 0.091, whereas the microstate type × group interaction did not reach statistical significance, *F*_(3, 114)_ = 2.44, *p* = 0.068, η^2^_*p*_ = 0.060. Neither the time × microstate type interaction nor the three-way interaction among Time, microstate type, and group was significant (both *p* > 0.20). For between-subject effects, a significant main effect of Group was observed when averaged across all conditions, *F*_(1, 38)_ = 4.67, *p* = 0.037, η^2^_*p*_ = 0.109, with estimated marginal means indicating higher overall contribution values in the IA group [mean = 25.27, SE = 0.09, 95% CI (25.09, 25.45)] compared with the HC group [mean = 25.00, SE = 0.09, 95% CI (24.82, 25.18)]. Follow-up comparisons showed (Figure 2A) that microstate D contribution was significantly lower in the IA group than in the HC group at baseline, *t*_(38)_ = 3.14, *p* = 0.006, whereas within the IA group, microstate D contribution increased significantly following the intervention, *t*_(19)_ = −3.71, *p* = 0.001. After the intervention, no significant between-group differences in microstate D contribution were observed.

### 3.3 Transition probabilities of EEG microstates

**Figure 3:**
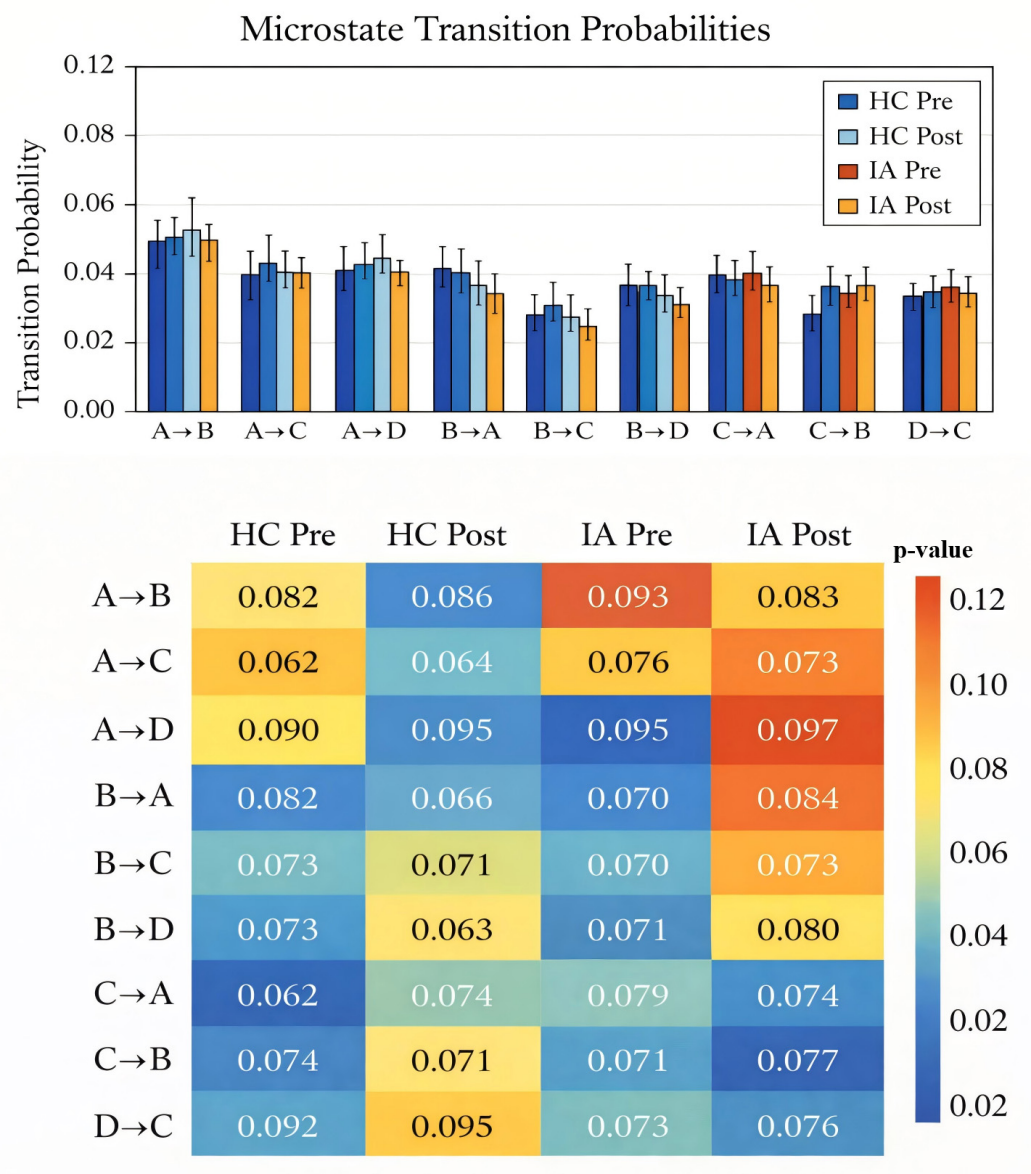
Effects of acute aerobic exercise on EEG microstate transition probabilities in internet addiction.

A mixed-design ANOVA with time (pre vs. post) and transition type (three levels) as within-subject factors and Group (HC vs. IA) as a between-subject factor was conducted for transition probabilities originating from each microstate (A–D). The result is shown in Figure 3. For transitions originating from Microstate A, no significant main effect of Time was observed [*F*_(1, 38)_ = 0.002, *p* = 0.963, η^2^_*p*_ < 0.001], nor was the time × group interaction significant [*F*_(1, 38)_ = 0.98, *p* = 0.329, η^2^_*p*_ = 0.025]. In contrast, a main effect of transition type emerged [*F*_(2, 76)_ = 9.93, *p* < 0.001, η^2^ = 0.207]. No other interaction effects reached significance (all *p* > 0.74), and the main effect of group was not significant [*F*_(1, 38)_ = 2.10, *p* = 0.155, η^2^_*p*_ = 0.052]. For transitions originating from Microstate B, the main effect of time [*F*_(1, 38)_ = 0.06, *p* = 0.813, η^2^_*p*_ = 0.001] and the time × group interaction [*F*_(1, 38)_ = 0.41, p = 0.528, η^2^_*p*_ = 0.011] were not significant. However, a significant main effect of transition type was observed [*F*_(2, 76)_ = 3.31, *p* = 0.042, η^2^ = 0.080], accompanied by a significant main effect of group [*F*_(138)_ = 4.54, *p* = 0.040, η^2^ = 0.107]. No significant time-related interactions were detected (all *p* > 0.33). For transitions originating from Microstate C, no significant main effects of time [*F*_(1, 38)_ = 0.02, *p* = 0.901, η^2^_*p*_ < 0.001], transition type [*F*_(276)_ = 0.80, *p* = 0.454, η^2^_*p*_ = 0.021], or group [*F*_(138)_ = 0.57, *p* = 0.454, η^2^ = 0.015] were observed. In addition, none of the interaction effects reached statistical significance (all *p* > 0.71), indicating stable transition patterns from Microstate C across time and groups, with no detectable modulation by acute exercise. For transitions originating from Microstate D, the main effect of time was not significant [*F*_(1, 38)_ = 0.37, *p* = 0.549, η^2^_*p*_ = 0.010], nor was the time × group interaction [*F*_(1, 38)_ = 0.87, *p* = 0.356, η^2^_*p*_ = 0.022]. However, a significant main effect of transition type was found [*F*_(2, 76_) = 5.83, *p* = 0.004, η^2^ = 0.133]. Although the transition type × group interaction did not reach statistical significance [*F*_(2, 76)_ = 2.73, *p* = 0.072, η^2^ = 0.067] No higher-order interactions were observed (all *p* > 0.78), and the main effect of Group remained non-significant [*F*_(1, 38) =_ 3.19, *p* = 0.082, η^2^ = 0.077].

### 3.4 Correlation between exercise-induced changes in microstate parameters and internet addiction severity

**Figure 4:**
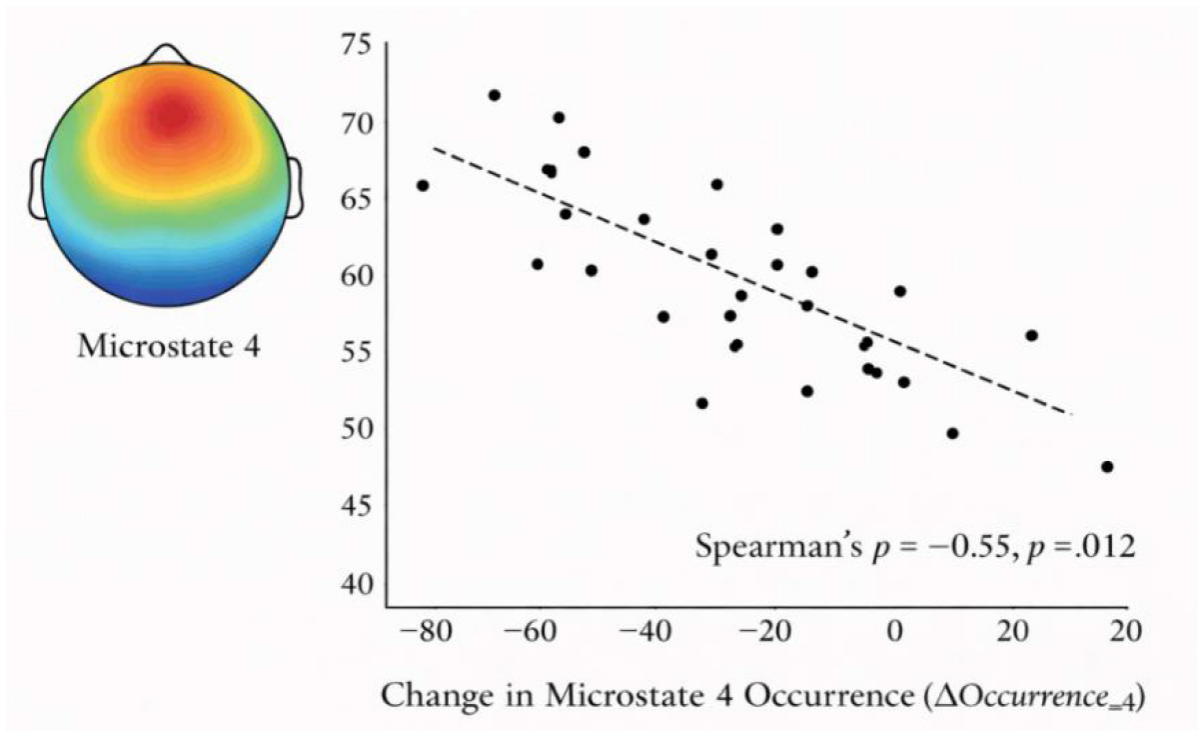
Correlation between changes in microstate D occurrence and IA scores.

Spearman's rank correlation analyses were conducted to examine the associations between intervention-induced changes in EEG microstate parameters (post-intervention minus pre-intervention) and internet addiction scores (n = 20). The results revealed a significant negative correlation between changes in the occurrence of Microstate D and IA scores (ρ = −0.55, *p* = 0.012), indicating that individuals with higher levels of Internet addiction showed attenuated increases or greater reductions in Microstate D occurrence following the intervention. In contrast, changes in microstate duration were not significantly correlated with IA scores (Duration_A: ρ = 0.12, *p* = 0.622; Duration_B: ρ = −0.07, *p* = 0.760; Duration_C: ρ = 0.10, *p* = 0.663; Duration_D: ρ = 0.03, *p* = 0.917). Similarly, no significant associations were observed between IA scores and changes in other microstate occurrence parameters (Occurrence_A: ρ = 0.05, *p* = 0.820; Occurrence_B: ρ = −0.05, *p* = 0.825; Occurrence_C: ρ = 0.06, *p* = 0.813). Changes in microstate contribution parameters were also not significantly related to IA severity (Contribution_A: ρ = 0.09, *p* = 0.719; Contribution_B: ρ = −0.02, *p* = 0.940; Contribution_C: ρ = 0.08, *p* = 0.726; Contribution_D: ρ = −0.18, *p* = 0.438). Taken together, these findings suggest that exercise-related modulation of Microstate D occurrence is selectively associated with individual differences in internet addiction severity, whereas changes in other microstate parameters appear to be largely independent of IA levels (Figure 4).

## 4 Discussion

The present study investigated resting-state EEG microstate dynamics in individuals with Internet addiction and examined whether a single bout of moderate-intensity aerobic exercise could acutely modulate these neural signatures. Several novel and theoretically meaningful findings emerged. First, individuals with Internet addiction exhibited selective abnormalities in microstate temporal parameters at baseline, most prominently reflected by prolonged duration of microstate A and reduced occurrence and contribution of microstate D. Second, although overall microstate duration was largely stable across time, acute aerobic exercise selectively increased the occurrence and contribution of microstate D in the Internet addiction group, normalizing these parameters toward the level observed in healthy controls. Third, exercise-induced changes in microstate D occurrence were negatively associated with Internet addiction severity, suggesting that individuals with more severe addictive symptoms showed blunted microstate modulation. Together, these findings provide converging evidence that Internet addiction is characterized by altered large-scale brain network dynamics and that a single session of aerobic exercise can rapidly reshape specific dysfunctional microstate patterns (Michel and Koenig, 2018; Britz et al., 2010; Asha et al., 2024).

At baseline, the Internet addiction group demonstrated a significantly longer duration of Microstate A compared with healthy controls. Microstate A has been consistently linked to lateralized auditory–language and phonological processing networks, with sources involving superior temporal and perisylvian regions (Choi et al., 2013; Dong and Potenza, 2014). Prolonged microstate duration is generally interpreted as increased temporal stability or reduced flexibility of the corresponding network (da Cruz et al., 2020). In the context of Internet addiction, extended Microstate A duration may reflect excessive internal verbal processing, rumination, or stimulus-driven reactivity, consistent with previous EEG findings of increased low-frequency activity and impaired cognitive control in addicted individuals (Khanna et al., 2015; Tomescu et al., 2014). Similar prolongation of specific microstates has been reported in substance use disorders and behavioral addictions, suggesting that reduced microstate switching may represent a shared neurophysiological trait of impaired adaptive control (Koenig et al., 2005).

More critically, the internet addiction group showed markedly reduced occurrence and contribution of Microstate D at baseline. Microstate D is widely associated with the frontoparietal executive control network, including dorsolateral prefrontal and posterior parietal cortices, and is thought to support attentional allocation, working memory, and cognitive flexibility (Kindler et al., 2011). Reduced Microstate D expression has been reported in conditions characterized by deficient executive control, such as attention-deficit/hyperactivity disorder, schizophrenia, and substance dependence (Mandolesi et al., 2018; Chang et al., 2012; Dietrich and Audiffren, 2011). Our findings extend this literature by demonstrating that Internet addiction is similarly associated with diminished engagement of executive-control-related microstates during rest, providing direct electrophysiological evidence for impaired top-down regulation in this population.

Importantly, although microstate duration parameters were relatively resistant to acute modulation, aerobic exercise induced robust changes in microstate occurrence and contribution, particularly for Microstate D, in the Internet addiction group. Following exercise, Microstate D occurrence and contribution increased significantly in the addicted group and no longer differed from healthy controls. This pattern suggests a rapid enhancement of frontoparietal network engagement after a single bout of aerobic exercise. Acute exercise has been shown to transiently increase prefrontal oxygenation, enhance catecholaminergic transmission, and improve large-scale network efficiency (Li et al., 2014; Di Lorito et al., 2021; Khoo et al., 2024). From a microstate perspective, these neurochemical and hemodynamic changes may facilitate more frequent recruitment of executive control networks, reflected as increased Microstate D expression. Similar exercise-induced normalization effects have been reported in resting-state functional connectivity and EEG spectral indices, but the present study is among the first to demonstrate such effects at the level of fast brain-state dynamics (Goldstein and Volkow, 2011; Volkow et al., 2016).

The transition probability analyses further indicated that differences across transition types were primarily driven by microstate-specific properties rather than time-dependent changes, with limited evidence for exercise-induced alterations in global transition structure. This relative stability of transition architecture is consistent with recent work suggesting that microstate transition patterns may reflect trait-like network organization, whereas occurrence and contribution are more sensitive to short-term neuromodulatory influences (Weinstein et al., 2017). Thus, acute aerobic exercise may preferentially influence the “amount” of time the brain spends in specific functional states, rather than fundamentally reorganizing state-to-state transition rules. Crucially, the correlation analyses revealed that exercise-induced increases in Microstate D occurrence were negatively associated with Internet addiction severity. Individuals with higher addiction scores showed attenuated microstate modulation, suggesting reduced neural plasticity or diminished responsiveness of executive networks to acute exercise. This finding aligns with behavioral and neuroimaging evidence indicating that greater addiction severity is associated with reduced prefrontal adaptability and impaired neuromodulatory capacity (Ko et al., 2013; Brand et al., 2014). From a clinical perspective, this result implies that although acute exercise can beneficially modulate brain dynamics, individuals with more severe addiction may require higher exercise doses, repeated sessions, or combined interventions to achieve comparable neural effects.

Several aspects of the present findings can be better understood in relation to earlier work. Previous neuroimaging studies of IA and related behavioral addictions have consistently reported abnormalities in brain systems responsible for executive control and attentional regulation, particularly in the dorsolateral prefrontal cortex, frontal regions, parietal regions, the anterior cingulate cortex, and the insula. Recent resting-state studies in Internet gaming disorder have further shown altered organization of the executive control network and the dorsal attention network, and these abnormalities have been linked to heightened impulsivity and impaired cognitive control (León Méndez et al., 2024). Within this broader framework, the reduced occurrence and contribution of Microstate D observed in the present study are consistent with the interpretation that Internet addiction is characterized by weaker recruitment of control-related large-scale brain states during rest. More specifically, these findings suggest that such control-related states occurred less frequently and occupied a smaller proportion of total resting time in individuals with Internet addiction (Zhao et al., 2024).

At the microstate level, the present baseline pattern partly converges with, but is not identical to, previous findings in Internet-related addictive behaviors. A previous study of Internet addiction disorder reported reduced duration and coverage of Microstate C and linked these abnormalities to impaired inhibitory control (Qi et al., 2023). In contrast, the present study identified prolonged duration of Microstate A together with reduced occurrence and contribution of Microstate D. Rather than contradicting earlier work, this difference may reflect variation in sample characteristics, diagnostic definitions, and the specific temporal parameters examined. It may also suggest that Internet addiction involves abnormalities across multiple control-relevant resting-state brain states rather than a single canonical microstate subtype. In this sense, the present findings extend earlier work by showing that the abnormality in Internet addiction may be reflected not only in how long a brain state is maintained, but also in how frequently a control-related state is recruited and how much total time it occupies. Consistent with the 2024 comparative study of addictive disorders, the present results further support the view that addiction-related abnormalities in brain dynamics are state-specific rather than reflected in a uniform shift across all microstate parameters (Kim et al., 2024).

The exercise-related findings can also be interpreted in light of previous research. Prior resting-state functional magnetic resonance imaging studies have shown that a single bout of moderate-intensity aerobic exercise can increase synchrony among regions involved in attention and executive control. Extending this literature, the present findings suggest that exercise-related modulation may also be detectable at the sub-second level of electroencephalography microstate dynamics, specifically as a greater temporal expression of control-related Microstate D in individuals with Internet addiction. In other words, acute aerobic exercise may not necessarily alter all resting-state brain-state parameters, but it may selectively enhance the recruitment of those states most closely related to executive regulation.

Differences between the present findings and earlier reports are not unexpected and may reflect heterogeneity in diagnostic definitions, analytical level, and temporal metrics examined. For example, earlier studies have variously focused on Internet addiction, Internet addiction disorder, or Internet gaming disorder, and have examined either resting-state functional connectivity or electroencephalography microstates, as well as different parameters such as duration, occurrence, contribution, coverage, or transition probability. Accordingly, the current results should be interpreted as complementing rather than replacing prior findings.

Overall, the present findings support a model in which internet addiction is characterized by an imbalance between over-stable stimulus-driven networks and under-recruited executive control networks at rest. Acute aerobic exercise appears capable of transiently restoring this balance by selectively enhancing Microstate D dynamics, thereby increasing the brain's readiness for cognitive control. These results provide a novel microstate-based neurophysiological mechanism for the beneficial effects of exercise in behavioral addiction and highlight EEG microstates as sensitive biomarkers for rapid, non-pharmacological neuromodulation (Schuch et al., 2016).

Several limitations should be acknowledged. First, the absence of a time-matched non-exercise control condition prevents strong causal attribution of the observed pre–post microstate changes specifically to aerobic exercise, as alternative explanations such as passage of time, habituation, non-specific arousal, order effects, or regression to the mean cannot be fully excluded. Nevertheless, because both groups underwent the same recording schedule and exercise protocol, the design still allowed comparison of short-term neurophysiological response patterns under a common intervention framework. Second, the sample size was modest, and no *a priori* power analysis was conducted, limiting confidence in the statistical sensitivity of the study, particularly for small-to-moderate effects. Third, although multiple-comparison correction was applied to *post hoc* analyses, some microstate-specific follow-up findings were not fully supported by omnibus interaction effects and therefore should be interpreted as exploratory. Future studies should adopt larger and better-powered samples, include sedentary control conditions, and combine electroencephalography with source localization or functional magnetic resonance imaging to clarify the mechanisms underlying exercise-related microstate changes.

## 5 Conclusion

This study demonstrates that Internet addiction is associated with selective alterations in resting-state EEG microstate dynamics, particularly involving Microstate A and Microstate D. A single bout of moderate-intensity aerobic exercise effectively increased the occurrence and contribution of Microstate D in individuals with Internet addiction, normalizing these parameters to levels comparable with healthy controls. Importantly, exercise-induced changes in Microstate D occurrence were negatively correlated with addiction severity, indicating individual differences in neural responsiveness to exercise. These findings suggest that acute aerobic exercise can rapidly modulate dysfunctional large-scale brain dynamics in internet addiction and support its potential as a non-pharmacological neuromodulatory intervention.

## Data Availability

The raw data supporting the conclusions of this article will be made available by the authors, without undue reservation.
